# Transparent Sol–Gel-Based Coatings Reflecting Heat Radiation in the Near Infrared

**DOI:** 10.3390/gels9100795

**Published:** 2023-10-02

**Authors:** Thomas Mayer-Gall, Leonie Kamps, Thomas Straube, Jochen S. Gutmann, Torsten Textor

**Affiliations:** 1 Deutsches Textilforschungszentrum Nord-West gGmbH, 47798 Krefeld, Germany; mayer-gall@dtnw.de (T.M.-G.); leonie.kamps@dtnw.de (L.K.); jochen.gutmann@dtnw.de (J.S.G.); 2 Center of Nanointegration Duisburg-Essen, University of Duisburg-Essen, 47057 Duisburg, Germany; 3 Mehler Texnologies—Freudenberg Performance Materials, 41836 Hückelhoven, Germany; thomas.straube@freudenberg-pm.com; 4 TEXOVERSUM School of Textiles, Reutlingen University, Alteburg Str. 150, 72762 Reutlingen, Germany; 5 RRI—Reutlingen Research Institute, Reutlingen University, 72762 Reutlingen, Germany

**Keywords:** architectural textiles, sol–gel technique, heat reflection, low-e

## Abstract

Thin, flat textile roofing offers negligible heat insulation. In warm areas, such roofing membranes are therefore equipped with metallized surfaces to reflect solar heat radiation, thus reducing the warming inside a textile building. Heat reflection effects achieved by metallic coatings are always accompanied by shading effects as the metals are non-transparent for visible light (VIS). Transparent conductive oxides (TCOs) are transparent for VIS and are able to reflect heat radiation in the infrared. TCOs are, e.g., widely used in the display industry. To achieve the perfect coatings needed for electronic devices, these are commonly applied using costly vacuum processes at high temperatures. Vacuum processes, on account of the high costs involved and high processing temperatures, are obstructive for an application involving textiles. Accepting that heat-reflecting textile membranes demand less perfect coatings, a wet chemical approach has been followed here when producing transparent heat-reflecting coatings. Commercially available TCOs were employed as colloidal dispersions or nanopowders to prepare sol–gel-based coating systems. Such coatings were applied to textile membranes as used for architectural textiles using simple coating techniques and at moderate curing temperatures not exceeding 130 °C. The coatings achieved about 90% transmission in the VIS spectrum and reduced near-infrared transmission (at about 2.5 µm) to nearly zero while reflecting up to 25% of that radiation. Up to 35% reflection has been realized in the far infrared, and emissivity values down to ε = 0.5777 have been measured.

## 1. Introduction

In recent years, textile architecture has become a growing business sector. Thin and lightweight textile membranes, e.g., coated fabrics, offer far-reaching creative possibilities in architecture and could be used in innovative building materials. At the same time, however, environmentally friendly, especially energy-saving, materials are of increasing importance. In this context, a major drawback of textile membranes is their negligible heat insulation properties compared to other modern building materials. A simple textile membrane will not achieve competitive thermal transmittance. Reduced thermal convection and conduction can only be achieved by combining textiles with, e.g., foams, via the use of three-dimensional fabrics or by producing inflatable membrane structures.

If one considers a building that has been constructed with a textile roof in hot and sunny surroundings, it is clear that thermal radiation will cause a significant contribution to heat build-up and thermal convection inside the building. This is why conventional roofs in such areas are often painted white and why textile roofing is often based on a metallized fabric or membranes. The metallic surfaces are able to reflect the heat radiation. As long as radiation is not absorbed by or transmitted through the roof, radiation energy will not be able to heat up the inside of such a building. This is the idea of a low-ε or low-emissivity material, which is a material that enhances energy efficiency in buildings by reflecting infrared energy and reducing heat transfer through a surface. Since the metallic coatings are not or are only slightly transparent, the metallization will always yield a mostly unwanted shading effect. If shading is a problem, it is solved by producing textiles with a more open surface or by patterning metallization. In that case, a reasonable compromise has to be found between reflectance for heat radiation and the transmittance of visible light. In that case, increasing the transmittance for VIS always yields a reduced reflectance of heat and vice versa.

In this regard, windowpanes, which combine high heat reflection and transparency for visible light, are commercially available [1]. Such windowpanes show high reflection in the near infrared above wavelengths of about 2 µm. They are coated either with very thin layers of silver or gold to achieve highly reflecting surfaces with a certain translucence or with very special metal oxides that are transparent for visible light. Such metal oxides are so-called transparent conductive oxides (TCOs) [2,3,4,5,6,7,8]. TCOs are typically based on semiconductive oxides with a broad band-gap that are n-doped with a comparably high share of donor atoms. The main applications of these TCO materials are electrode materials for displays [4,9,10] of flat screens, smartphones or electrodes of solar cells [11,12,13]. Above all, these products need transparent electrodes with a high electrical conductivity. Heat reflection appears to be an incidental outcome rather than a requirement for these applications. A well-known TCO showing the best performance with respect to conductivity and transparence is indium-doped tin oxide (ITO) [6,14,15,16,17]. There are also developments based on aluminum-doped zinc oxide (AZO) [18,19,20,21,22] or others that are produced of more available metals than indium [6,23,24,25,26,27]. The TCO coatings for all these applications are applied by vacuum techniques demanding pure atmospheres and often temperatures of 450 °C and more [28,29]. A lot of these techniques allow the production of very thin and precise coatings free from any defects as needed for displays or solar cells. On the one hand, defects would disturb the visual impression and the quality of a display. On the other hand, they affect the electric properties in an inadmissible way or lead to short-circuits.

TCOs should also be an ideal material for producing heat-reflecting transparent textile membranes. In a review paper, Pasquarelli et al. [29] mention a number of TCOs and the elaborate application techniques, but also discuss various solution-based TCO coatings. Even though vacuum techniques, especially low-pressure, low-temperature plasma treatment, etc., have been discussed for textile modification in research and literature for decades [30,31,32], there has been no actual breakthrough. Even if a transfer and scale-up to textile processing would be successful, vacuum processes are costly and those required for depositing TCOs are mostly accompanied by high process temperatures. The application from solution bears a lot of advantages in view of textile application. However, the reported application processes mostly employ annealing temperatures above 300 °C, some even higher than 1000 °C, which are prohibitive for nearly all textile materials.

Applications of TCO coatings have not been industrially successful so far. The major factor is the detrimental effect on product quality with respect to conductivity, which is connected to the annealing temperatures. Here, it has to be taken into account that solar-cell research or display developments are focused on improved efficiencies, e.g., to raise the visible transparency from 98 to 99% or to minimize the electrical resistance. If such superlatives are not necessary, as is the case with heat insulation of textile membranes, compromises become possible.

The coating of a textile membrane would not need to be electrically conductive nor be free of microdefects to provide reflective properties for heat radiation, at least not at a comparable level to that of an electronic device. Therefore, wet-chemical approaches should be of interest in this area. Highly crystalline TCO materials based on ITO, AZO or antimony-doped tin oxide (ATO) are available as fine powders or dispersions. As Aegerter et al. [33,34] showed, it is possible to produce highly transparent and moderately conductive coatings by preparing dispersions of the functional particles combined with certain amounts of sol–gel-based binder chemistry. While in the review from Pasquarelli et al. [29] annealing after coating at very high temperatures is described, Aegerter et al. [34] only cured at about 130 °C combined with a UV treatment. To improve resistance of the resulting films, Aegerter et al. carried out an annealing at 130 °C in a reducing atmosphere (forming gas) for 20 h. Furthermore, in our earlier research focused on synthesizing AZO nanoparticles for membrane coatings, we noted that, as conductivity increases, there is a corresponding increase in IR reflection [22,35].

Sol–gel-based coatings for textile materials have been investigated since the late 1990s as a versatile approach to achieve numerous finishing effects [36,37]. Sol–gel-based coatings are basically inorganic–organic hybrid polymers that can, e.g., act as a binder for fillers as nanoparticles. Starting from this consideration, the focus of this work was to investigate whether it is possible to follow a comparable approach to apply a transparent and heat-reflecting coating to textile membranes by employing simple techniques available for the producers of technical textiles. Coating “lacquers” based on TCO nanoparticles and sol–gel-based binders were synthesized. The colloidal solutions were first coated onto polyester films to investigate the coatings and their properties in more detail. The focus was on appearance, transparency, absorption and reflectance for visible light as well as for infrared or heat radiation. The topography of the coatings has been investigated looking for distribution of the TCO particles as well as homogeneity and thickness of the coatings. Selected systems were applied to technical samples of textile membranes. Coatings were also characterized and the heat-reflecting effect was also tested with an IR camera. As the focus has been on a simple process adaptable for the textile industry, only a comparably short curing period at 130 °C in air has been chosen.

## 2. Results and Discussion

### 2.1. Preparation and Characterization of the TCO Dispersions

The three commercial TCO systems were tested in a first set of experiments in view of stability and particle size, which should be small in view of optical transparency in the VIS. In case of the TCO dispersion, processing was simple as the dispersion needed only to be mixed with additional solvents, silanes and catalyst (hydrochloric acid). The powders, in contrast, have to be dispersed in an additional processing step using a sonotrode. Tests were carried out with different dispersant media such as water, ethanol, isopropanol or 2-methoxyethanol. Particle sizes were measured by DLS and for each material the dispersion yielding the smallest average particle size was chosen for further investigation. Table 1 presents the data of three dispersions of ITO and AZO, respectively, showing the smallest average particle sizes. 

While the average particle size in the ready-made ITO dispersion supplied by Sigma-Aldrich is smaller than 100 nm, the average particle size in the dispersions prepared from powders were 245 nm in the case of ITO and 1290 nm in the case of AZO. This must be compared to powder specifications of 50 nm for ITO (Sigma-Aldrich) and 20–40 nm for the AZO powder (IoLitec). Dispersions of both TCO materials, which were prepared with other dispersants as presented in Table 1, were either very unstable or exhibited bigger particles sizes. All dispersions chosen for preparation of coatings were stable enough to allow a timely processing and application. At this stage, no extensive optimization of the dispersing process was tested.

### 2.2. Preparation of Coatings

Further processing of the chosen dispersion was conducted to identify the system with promising performance in view of suitability for coating textile roofing membranes in a comparably simple approach and their optical and heat-insulating properties.

**Figure 1:**
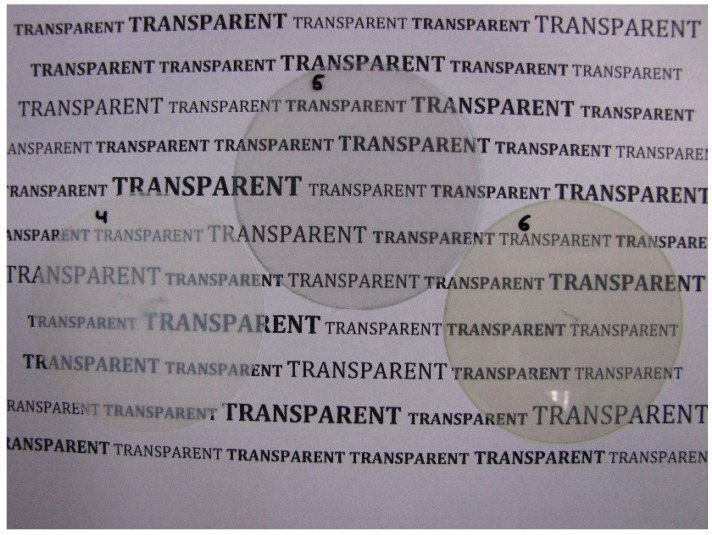
PET films spin-coated with three different TCO coatings. The left sample (4) is prepared with the AZO particles, the one in the middle (5) with ITO particles delivered as dispersion and the right one (6) with ITO powder dispersed by ultrasonic treatment.

The chosen particle dispersions were mixed with an alkoxysilane-based sol–gel binder system. The binder system is prepared by dissolving 3-(trimethoxysilyl)-propyl methacrylate (METHA) in alcohol and hydrolyzation by adding a stochiometric amount of hydrochloric acid. The addition of hydrochloric acid initiates the hydrolyzation and subsequent condensation of the silane, yielding a METHA-sol (hybrid polymer sol). The molar ratio of HCl to alkoxysilane is 3:2. The prepared binder is stirred for 1 h before it is mixed with the particle dispersion. More details can be found in the Materials and Methods. The resulting mixtures were applied to PET films by spin-coating and cured at 130 °C for 30 min. The evaporation of the solvent and the heating accelerate the condensation reaction and enable crosslinking of the methacrylate groups, finally yielding a xerogel. Among others, Al-Dahoudi et al. [34] have described comparable sol–gel-based hybrid polymer systems. Differences in the investigations presented in this paper concern the relation between binder and TCO particles in the cured coatings. For the examples shown below, the share of TCO in the resulting coating is about 94 wt.%. The photographs in Figure 1 show the corresponding PET films that were coated as described. While the samples prepared with the ITO particles appear highly transparent, the one prepared with the AZO particles appears slightly turbid. This is in good agreement with the DLS measurements, which showed strongly agglomerated AZO particles in the dispersion. The sample coated with the ready-made ITO dispersion appears slightly blueish, the one prepared with the ITO powders slightly yellow, while the AZO sample shows no specific color shade.

### 2.3. Comparison of Some Electrical and Optical Properties of Different TCO-Coated Samples

As explained in the Introduction section, pure TCO films are known to exhibit low surface resistance as well as a low emissivity. However, the major criteria of the studied TCO composite coatings in view of the application in textile architecture are transparency and reflection of heat radiation. Therefore, conductivity and surface resistance are not determining factors in the first place. Nevertheless, surface resistance is a good indicator for percolation of the conductive particles. Hence, it was of interest in the proceedings of the experiments to see whether there was an effect on reflection of heat radiation as well. The composite TCO coatings on PET film substrates were also investigated with respect to these values. The corresponding data are summarized in Table 2.

As expected, the sheet resistance of the pristine film was very high and no value could be measured. The same was true for the sample that was coated with the composite containing AZO particles. Consequently, no surface conductivity worth mentioning can be observed for the AZO-based coating. Sheet resistances of both ITO-based samples were 330 mOhm/sq.

The emissivity of the PET film showed a typical value for organic polymer surfaces with ε = 0.8695 [38,39]. The known low emissivity of pure TCO films could be measured for composite coatings as well, which all reduced the emissivity of the coated PET film. In the case of the AZO-based coating, the reduction of the emissivity was comparably low, while it ranged from 19–22% for the ITO systems.

**Figure 2:**
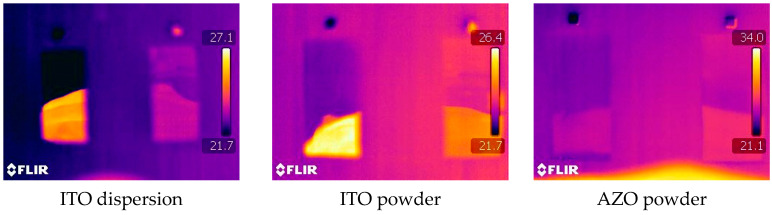
Three thermograms, each comparing the heat reflection of a coated PET sample (**left**) and uncoated PET stripe (**right**). One can observe the reflection of the heat of a human hand.

The differences in the emissivity could also be visualized using an IR camera recording uncoated and coated PET samples simultaneously while a hand is held in front of the samples. In each thermogram in Figure 2, the sample with TCO coating is on the left and the uncoated sample on the right side. Both ITO-coated samples clearly exhibit strong reflection of heat radiation stemming from the person’s hand, while the AZO sample shows no distinct improvement.

### 2.4. Influence of the ITO Share for Optical and Electrical Properties

As the AZO-based dispersion and coating, respectively, were not promising in view of optical transparency and heat reflection, further investigation was carried out with ITO only. The ready-made dispersion was chosen, as the preparation of the coating sol is less elaborate and the dispersing process can be left out. Furthermore, the chosen ITO dispersion exhibited the smallest average particle size, which can be expected to guarantee low light scattering in the VIS area.

**Figure 3:**
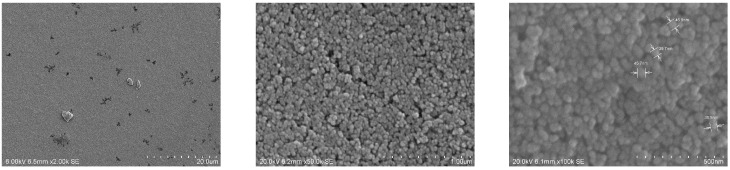
SEM micrographs at different magnifications of an ITO-based coating using METHA as a sol–gel binder. The share of ITO in this coating is about 94 wt.%. The right micrograph shows results for the measurement of particle size of some particles. Diameters of the four particles vary from 38.5 nm to 46.8 nm. (Due to the software, the scale in the micrographs is indicated as “um”, which stands for micrometers (µm).).

**Figure 4:**
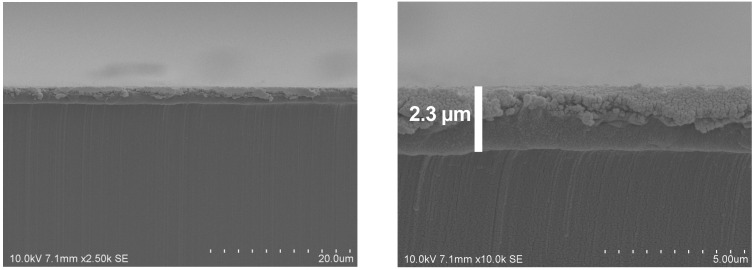
SEM micrographs showing a cross-section of the PET film coated with the TCO composite at different magnifications (94 wt.% ITO). (Due to the software, the scale in the micrographs is indicated as “um”, which stands for micrometers (µm).).

The coatings produced with the ITO dispersion were investigated by SEM in more detail. SEM micrographs of low magnification show flat homogeneous surfaces (cp. Figure 3, left). At high magnification, one can observe the particles forming the coating on the nanoscale. Measurements of the particle size that can be observed in the SEM show diameters smaller than 50 nm, which shows that no agglomeration worth mentioning occurs in the dispersion employed for coating. The diameters observed are in the range of the primary particle size as specified by the supplier. The coating shown here contains a share of 94 wt.% ITO. The corresponding SEM micrographs are depicted in Figure 3. Figure 4 shows micrographs with two different magnifications of a cross-section of the coated film. Coating appears homogenous and a thickness of about 2.3 µm can be roughly estimated.

**Figure 5:**
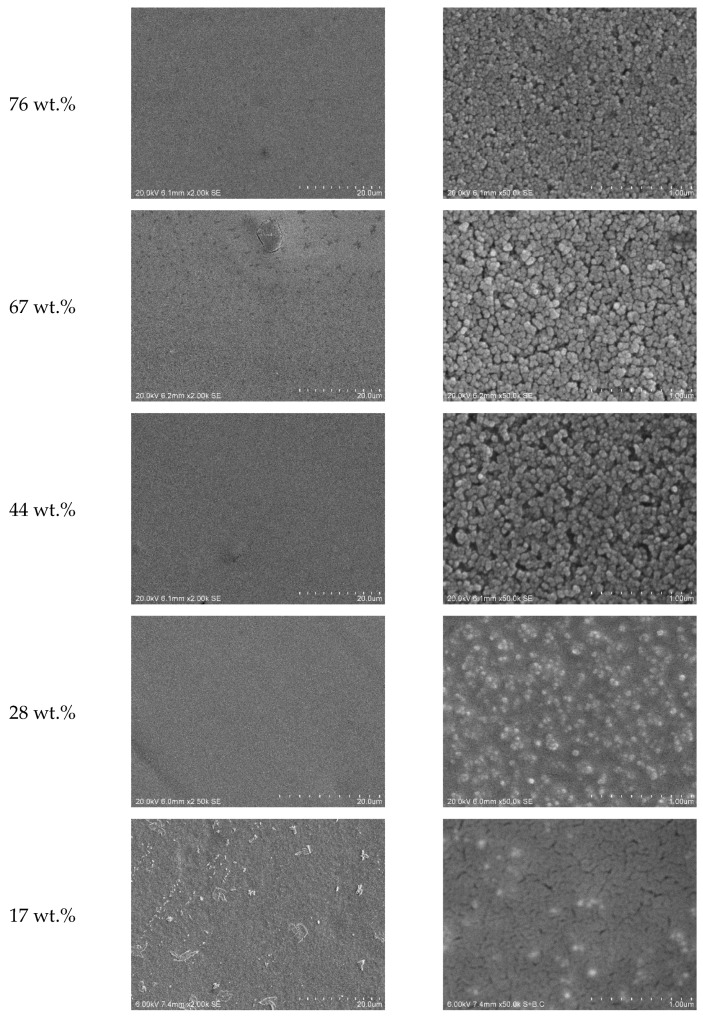
SEM micrographs of ITO-loaded sol–gel coatings with different shares of ITO. The left column shows an overview over the coatings while the right column shows high-magnification micrographs that show the distribution of the ITO particles within the coating. (Due to the software, the scale in the micrographs is indicated as “um”, which stands for micrometers (µm).)

The coatings with the high share of ITO appear comparably dense. It appears as a more or less dense layer of particles only, while the presence of the binder is not visible. The following SEM micrographs (cf. Figure 5) show a series of coatings with increasing share of the binder in the receipt. At a concentration of only 44 wt.% ITO, it can be observed that the coating seems to be less dense with more or bigger cavities between the particles. As the share of particles decreases to 28 wt.%, an obviously continuous binder film with embedded particles can be observed.

**Figure 6:**
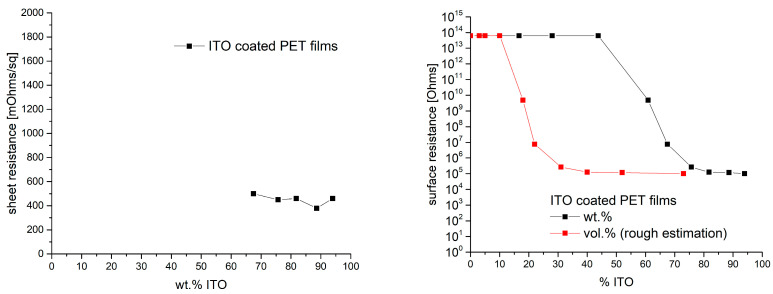
Sheet resistance of PET films coated with ITO composites of different ITO loads. For loads with less than 67 wt.% ITO, no values were measurable with the set-up available (**left** graph). Surface resistance values measured according to DIN EN 1149-1 for the same set of samples (**right**).

Following the SEM analysis, the samples were characterized in view of electrical conductivity. A sheet resistance could only be measured for those samples with an ITO load in the composite coating of at least 67 wt.%. Sheet resistances were in the range between 380 and 500 mOhms/sq (cf. Figure 6, left). Sheet resistances for samples with lower ITO load were not within the measuring range. To somehow present the dependence of the resistance on the ITO load at lower concentrations, the surface resistance according to DIN EN 1149-1 was measured in addition to the sheet resistance. Very high resistances of ≥10^14^ Ohms were measured for coatings with up to 44 wt.% ITO. Higher concentrations lead to a drop in the surface resistance. Concentrations of 67 wt.% and higher yield surface resistances of about 10^5^ Ohms. The corresponding data are summarized in Figure 6.

As density values for neither particles nor the cured binder were available, working with wt.% seemed to be straightforward. Just to make a rough calculation and to get an idea, we estimated an ITO density of 7.12 g/cm^−3^ [40] and a binder density of about 1 g/cm^−3^, which suggests that approximately 17.5 vol.% ITO corresponds to 60 wt.% ITO. Accordingly, the observed strong decrease in the surface resistance between the samples with 40 wt.% ITO and 60 wt.% ITO is in good agreement with theoretical predictions first reported by Scher and Zallen in the 1960s [41,42], which assume that percolation of spherical particles can be expected at concentrations higher than 16 vol.%. In Figure 6 (right graph), the surface resistance versus the mentioned estimated volume fraction of ITO is exemplarily shown as well. In this presentation, the steep drop in the resistance due to the exceeding of percolation threshold is more obvious.

**Figure 7:**
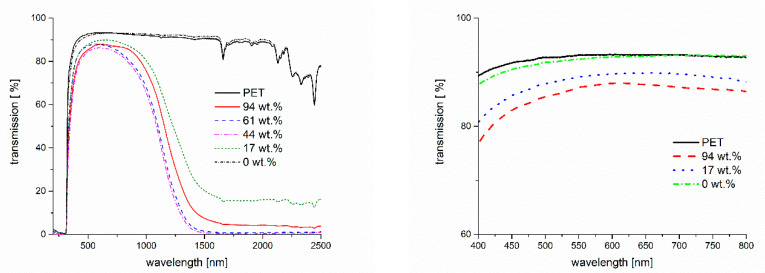
UV-VIS-NIR transmission spectra of PET films coated with ITO composites with varying ITO shares. The **left** spectrum shows the complete spectra while the **right** one illustrates the VIS area only. Note: 0 wt.% refers to the pure binder.

Transmission spectra of the coated films are shown in Figure 7. The UV-VIS-NIR spectra show that the binder itself does not influence the transmission spectrum compared to the pristine PET. Adding the ITO particles to the coating reduces transmission in the VIS area to a certain extent but, even in the case of adding 94 wt.%, the transmission remains between 80% and 88% (cp. Figure 7, right). The transmission of the pristine PET film is up to 93%. At the same time, the addition of the ITO particles to the binder results in a strongly reduced transmission at wavelengths longer than 800 nm. This reduction depends on the share of ITO in the applied coatings, i.e., the higher the share, the lower the transmission of NIR radiation.

**Figure 8:**
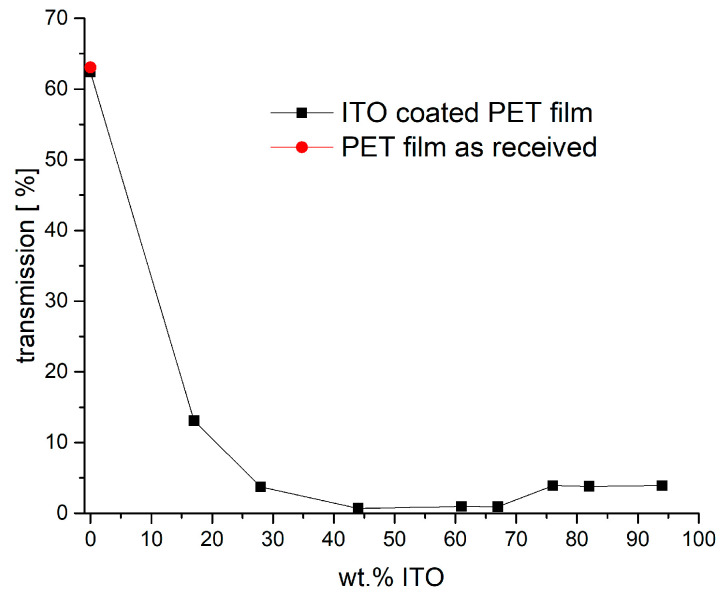
Transmission at a wavelength of 2.45 µm for PET films coated with ITO composites of varying ITO–binder ratio or ITO share, respectively. The red dot shows the value for the PET film without any coating.

To visualize the excellent blocking of heat radiation, Figure 8 shows the transmission at a wavelength of 2.45 µm in dependence of ITO share in the coating composite. It is clearly seen that the transmission drops steeply with addition of already low amounts of the ITO nanoparticles. A share of about 40 wt.% ITO in the composite already guarantees a nearly complete blocking of the radiation. A certain increase in transmission is observed here for weight fractions of 76% or higher. A possible explanation might be found in the coating morphologies as shown in Figure 5. A marked difference can be observed between the samples with 67 wt.% and 76 wt.% ITO. While the coating with lower particle load appears to be formed by clusters of individually connected particles, the sample with the highest load seems to be more homogenous and does not show big clusters. One may assume that this more homogenous and less rough coating reduces scattering, which explains the increased transmission here.

**Figure 9:**
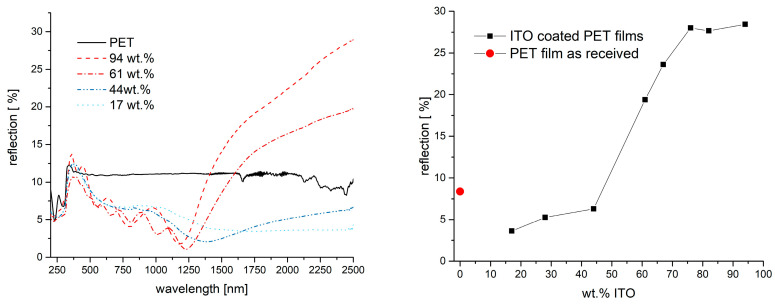
Reflection spectra of PET films coated with ITO composites made with different ITO–binder ratios (**left**). The **right** graph shows the reflection of the different samples at a wavelength of 2.5 µm. The red dot shows the corresponding reflection of the PET film without any coating.

While the transmission spectra show a uniform trend, the discussion of the reflection spectra is more complex. The corresponding reflection spectra are shown in Figure 9. For coated samples, the reflection in the range of visible light and NIR up to 1500 nm is lower compared to the reflection of the uncoated PET film. Especially in the VIS range, the reduced reflection seems not to be dependent on the share of ITO in the coating composite. The reflection is reduced more and more from about 400 nm to about 1200 nm. Above about 1200 nm, the reflection curve rises only in the case of those films that were coated with an ITO composite containing more than 44 wt.% ITO. In this spectral range, the reflection also depends on the ITO content of the coatings. Compared to the PET film, the coatings with ITO concentration of 61% and higher show a distinctly improved reflection in the NIR area at wavelengths higher than 1500 nm, while the coatings with less ITO show a distinctly lower reflection than the PET films. Coatings with 61 wt.% ITO yield a maximum reflection of 20% at 2.5 µm, while those with 67% yield 24%. Higher ITO shares yielded maximum reflection of about 28%.

When a material contains particles or components that can interact with IR radiation, percolation theory predicts that there will be a critical concentration at which the material starts to exhibit significant changes in its properties. At this point, the IR reflectivity will increase. Accordingly, correlation between the IR reflection and degree of particle connection are reported in papers by, e.g., Gao et al. [43] or Ederth et al. [44].

The onset of the rise of the reflection curve at about 1200 nm shows the so-called plasma edge. First of all, the position of this plasma edge depends on the specifications of TCO material (e.g., particle size, specific material, amount of dopant, lattice position of the dopant in the host crystal and many more). Reflection of infrared light increases for wavelengths longer than the plasma edge. The position of the plasma edge, therefore, determines in which regimes a TCO coating can improve the reflection. The theory of plasma edges will not be discussed in further detail here but can be found in a paper by Ruske et al. [41], who discuss the electron behavior of a highly doped ZnO. Besides the specific TCO material, the plasma edge and the reflectivity of a coating as discussed here also depend on the coating’s properties. A reduced roughness of the coating will increase the average density of charge carriers, thus increasing the reflectivity [45]. In agreement with this, the sample with 76 wt.% appears to be less rough and shows high reflection compared to the rougher ones with less particle load (cf. Figure 5). Additionally, the plasma edge is expected to shift to shorter wavelengths and, at the same time, the reflectivity increases with increasing volume fraction of the conductive particles, as reported, e.g., by Trollmann and Pucci [46,47]. The same behavior can be seen in Figure 9.

**Figure 10:**
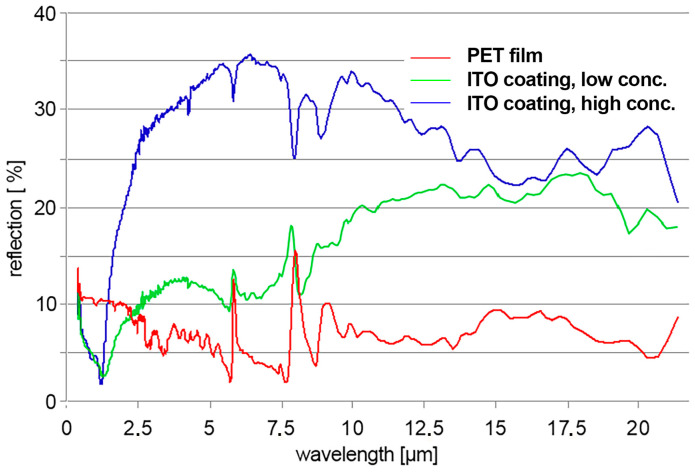
UV-VIS-IR spectra covering a wavelength range from 450 nm to 22.5 µm. The curves show the reflection of PET (line 1, red), an ITO coating with low TCO share (line 2, green) and a highly loaded composite (line 3, blue).

Within this work, the investigation mainly focused on the spectral range from 200 to 2500 nm as it is the range of solar radiation that is measured at the Earth’s surface. Exemplarily, measurements covering a wider range of infrared were conducted by an external laboratory. The reflection curves are shown in Figure 10. The spectra of PET, a coating with a lower amount of ITO and one with a high load are compared. While the coating with the lower ITO amount exhibits less reflection below 2.5 µm as already seen before, it exceeds the PET reflection at higher wavelengths. The highly loaded ITO coating shows a reflection between 25% and 35% in this range, which is distinctly higher than the reflection of the PET film (roughly between 5 and 10%). An increased reflection from 2.5 µm to 40 µm is relevant for reflection of body heat or to reflect heat and keep it, e.g., in a heated building, tent, etc.

**Figure 11:**
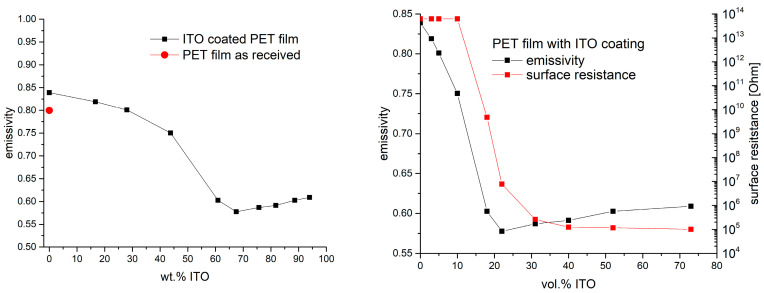
Emissivity of PET film and films coated with ITO composites with varying share of ITO in the binder. The red dot shows the corresponding reflection of the PET film without any coating. The graphs in the **right** figure show the already presented values for emissivity (cf. Figure 11 **left**) and surface resistance (cp. Figure 6, right) but plotted against the estimated ITO share in vol.%.

As stated earlier in the paper, it was of principal interest to see whether there was a certain correspondence between surface conductivity, heat reflection and emissivity. A comparison of the measured emissivity of the samples and surface conductivities (each in dependance of the ITO loading) is shown in Figure 11. The presented emissivities are determined by the employed spectrometer in the range from 2.5 µm to 40 µm. The behavior of the emissivity is qualitatively the same as for the reflection, which was discussed before. As long as the ITO share in the composite coating is 44% or lower, the emissivity of the coated films is roughly the same as for the PET film. Emissivity values are in a range between 0.85 and 0.75. Coatings that were prepared with a share of 61% ITO or more show a distinctly lower emissivity of around 0.6. In this case, variation in the emissivity for coatings is a maximum of 5%. The lowest value of all experiments here was ε = 0.5777. Figure 11 also shows the corresponding values of the surface resistance taken from Figure 6. When plotting the emissivity and surface resistance values against the share of ITO in vol.%, the connection between conductivity and emissivity becomes obvious. The steep drop in both values occurs in the region where percolation is expected. As mentioned above, the shares in volume percentage were only given as a rough estimate.

**Figure 12:**
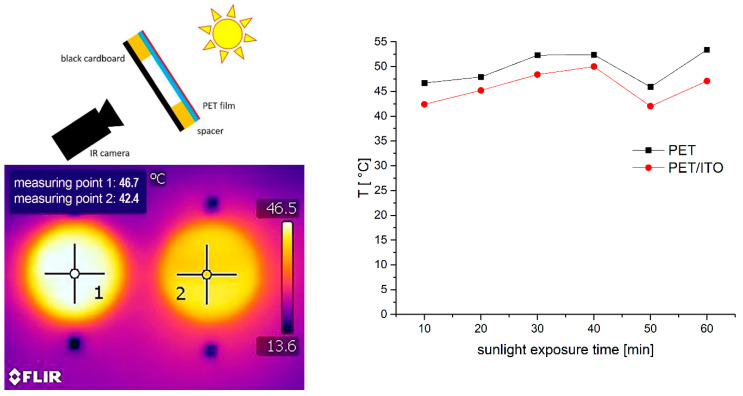
Sketch of the measuring configuration—the coating is directed to the Sun (**upper left**), exemplarily shown thermogram taken after 10 min (**lower left**) and the temperature course measured over 60 min (**right**).

As depicted in Figure 12, exemplary measurements were carried out with a simple setup to investigate differences in heat insulation while irradiating coated and uncoated PET films with actual sunlight. Black cardboard was positioned beneath the films and the temperature rise of the cardboard was measured as a function exposure time. The results show a fast heating of the cardboard from ambient temperature (~30 °C) to more than 40 °C. However, the sample covered with the coated PET film remains cooler over the test period of 60 min.

### 2.5. ITO Coatings on Textile Roofing Membranes

**Figure 13:**
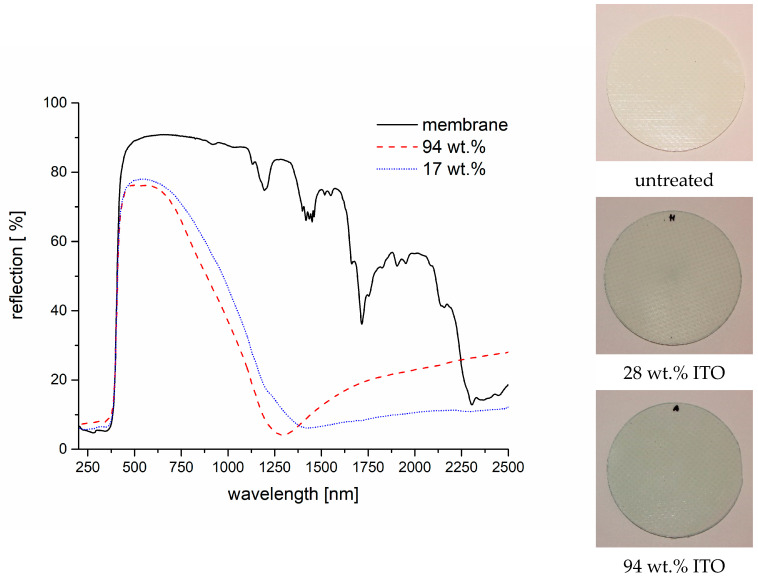
UV-VIS-NIR reflection spectra of a roofing membrane coated with ITO composites with an ITO share of 28 wt.% and 94 wt.%. The photographs at the right side show the untreated roofing membrane and those coated with the stated ITO composites.

So far, the investigations were conducted on PET films. With the aim of low-ε coatings for textile architecture ITO in mind, coatings were applied to textile roofing membranes as well. The substrate was a commercial, white PVC-coated roofing membrane. The photographs of the membranes in Figure 13 show that the blueish color mentioned at the beginning of this paper strongly influences the appearance if a white substrate is used instead of a transparent PET film. Even a coating with a load of only 28 wt.% ITO changes the appearance strongly. The general findings with regard to the reflection properties are as expected from the results discussed above. Corresponding data for the roofing membrane material can be seen in Figure 13. As the roofing membrane shows a much stronger reflection for VIS and NIR than the PET films, an improved reflection for NIR can only be observed for wavelengths longer than 2200 nm.

**Figure 14:**
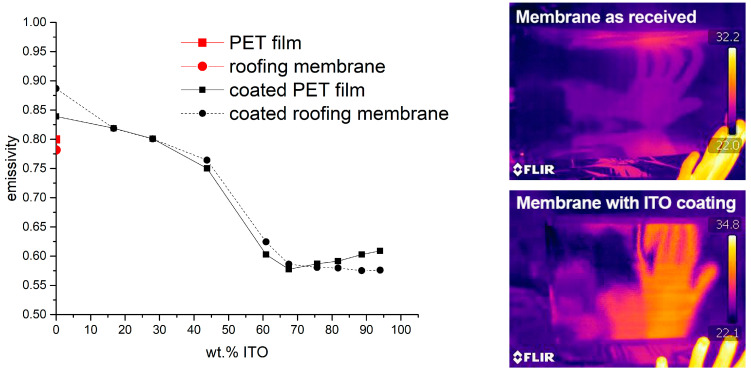
Emissivity of a PET film and a white roofing membrane coated with ITO composites with different ITO loading (**left**). The two photographs show thermograms of the membrane as received (**upper** thermogram) and the membrane with an ITO coating (thermogram **below**). The reflection of the body heat emitted by a human hand in front of the membrane is strongly increased for the coated sample.

Even though the reflection in the VIS-NIR area is downgraded, the improvements with respect to emissivity are completely comparable to what has been found for the PET films. Figure 14 shows the comparison of emissivity of PET films and roofing membrane coated with the same ITO composites. As mentioned above, emissivity is measured from 2.5–40 µm and, therefore, in the range that follows up the range of the UV-VIS-NIR spectra presented. Two thermograms are presented to visualize the heat reflectance. While the uncoated membrane (left photograph in Figure 14) shows only a slight heat reflection, the coated membrane reflects body heat (λpeak ≈ 9.5 µm) distinctly.

**Figure 15:**
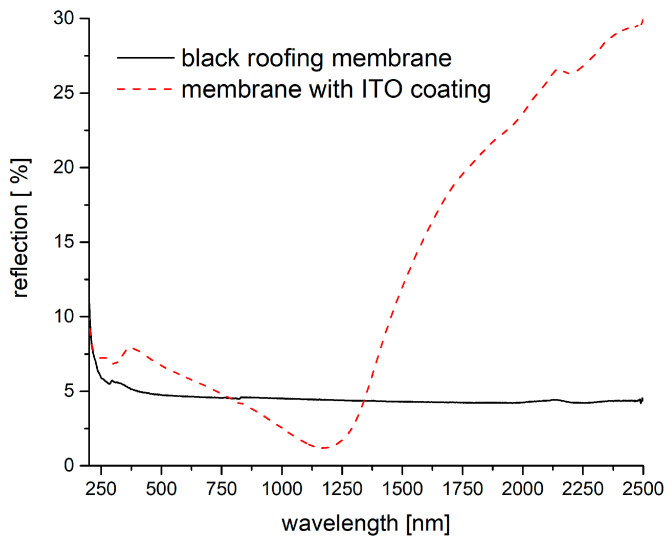
VIS-NIR spectra of a black roofing membrane and the corresponding membrane coated with a highly loaded ITO composite.

While the ITO coatings have a negative effect for the reflection of a white PVC-coated membrane in the VIS-NIR area and a positive effect for the IR area (>2.5 µm), they have a positive effect for the wavelength area starting at 1.4 µm in the case of a black membrane. In the case of a black membrane, TCO coating would improve the reflection of heat without affecting its appearance. Naturally, the black membrane has a low reflectivity in the VIS area, and the dyestuffs or pigments also absorb in the NIR as shown in Figure 15. Regarding the black roofing membrane, an improvement can be observed for wavelengths longer than 1.4 µm, and the influence in the VIS area is comparably small. The emissivity of the black membrane (measured from 2.5 µm to 40 µm) with the ITO coating (94 wt.%) is comparable to that of the white membrane and shows a value of ε = 0.65.

## 3. Conclusions

A comparably simple approach has been followed to apply transparent and heat-reflecting coatings on flat substrates aiming at the preparation of low-e coatings for textile architecture. Transparent conducting oxides are widely used due to their unique properties of being transparent in VIS, electrically conductive and heat reflecting. Sol–gel-based coatings loaded with TCO nanoparticles were prepared on the basis of commercially available TCO dispersion and powders. From these, one system based on a ready-made ITO dispersion was chosen for deeper investigation on the background of its optical and electrical properties as well as ease of preparation. Coatings prepared with different loads of ITO nanoparticles were applied to PET films and commercial roofing membrane. The results show that the derived coatings exhibited high transparency in the VIS spectrum. Even in the case of high ITO loads (94 wt.%), the transparency of the coated PET films was in a range between 80 and 88%. SEM micrographs showed homogenous coatings composed of the ITO nanoparticles and the binder. A cross-section exemplarily taken of a coating loaded with 94 wt.% of ITO indicated a layer thickness of 2.3 µm. The micrographs gave no evidence of bigger agglomerations. A very high blocking of heat radiation was found for all TCO-loaded coatings. Transmission of coatings loaded with only 17 wt.% of the ITO was lower than 20% for wavelengths between 800 nm and 2.5 µm. A nearly complete blocking of heat radiation in this wavelength range was observed for higher concentrations. In the case of an ITO load below 60 wt.%, the reduced transmission is attributed mainly to an increased absorbance as no increased heat reflection could be detected. In the case of highly loaded coatings, a distinctly increased reflection of NIR radiation with wavelengths above 1250 nm was measured. The highest heat reflection at 2.45 µm measured in this work was about 30%. Measurements of the coatings’ surface resistance showed that the distinct increase in reflection for coatings with a load higher than 60 wt.% corresponded to a steep drop in surface resistance. This steep drop indicates percolation. A rough estimation of the volume share of ITO at a 60 wt.% load gives approximately 16 vol.%, which is in good agreement with the percolation threshold expected for composites with spherical particles. Reflection measurements from 2.5 µm to 40 µm for highly loaded coatings showed a reflection in the range of 25% to 35% over the complete range of wavelengths. Emissivity of those samples was reduced to about ε = 0.6 compared to about ε = 0.8 for the PET films or a white roofing membrane. After coating, the white membrane showed a slightly blueish color, which is due to the color of the ITO particles employed. Nevertheless, the transparency of the coating was in the range discussed above. As expected, the TCO coating reduced the reflection of the white membrane in the VIS area and only improved reflection for the (N)IR radiation. Overall, the results show that the presented approach might be interesting for the development of transparent and heat-reflecting roofing membranes as well as for windowpanes. Future investigations planned by the authors will address aspects like durability, continuous coating processes as well as the preparation and use of cheaper TCO materials. Parallel to this work, some authors synthesized highly conductive AZO particles. A hydrothermal synthesis has been employed [22,35]. Bringing together results of the investigations presented here and the synthesis of the AZO particles might help to develop less expensive transparent low-e coatings that can be applied by simple coating techniques.

## 4. Materials and Methods

### 4.1. Materials

Three TCO materials have been tested: an indium-doped tin oxide (ITO) nanoparticle dispersion (30 wt.% in isopropanol) from Sigma-Aldrich, Taufkirchen, Germany, ITO nanoparticles as a powder from Sigma-Aldrich, Taufkirchen, Germany, and aluminum-doped zinc oxide (AZO) nanoparticles as a powder from IoLiTec, Heilbronn Germany. 3-(trimethoxysilyl) propyl methacrylate (METHA) (98%) was supplied by ABCR, Karlsruhe, Germany. Furthermore, the following solvents and auxiliaries were used: ethanol (96 vol.%, denatured with 1 vol.% butanone) supplied by Azelis, Krefeld, Germany, isopropanol (>99.5 vol.%), 2-methoxyethanol (>99%), hydrochloric acid (37 wt.%) supplied by Roth, Karlsruhe, Germany and water (deionized, 18.2 mOhms).

PET films used as substrates for the spin-coating experiments were conventional films as used for laser printers with a thickness of 100 µm (films obtained from Avery Zweckform, Oberlaindern, Germany). Roofing membranes were commercial white membranes based on PET fabrics coated with PVC and filled with titania. A black commercial facade membrane was used as an alternative to the white version.

### 4.2. Preparation

#### 4.2.1. Preparation of the Binder

A sol acting as a binder was produced by dissolving 10 mL METHA in 10 mL ethanol. The solution was thoroughly stirred using a magnetic stirrer before 1.08 mL and 0.1 molar hydrochloric acid were added. The mixture was stirred for at least one hour and subsequently processed.

#### 4.2.2. Preparation of TCO Dispersions

Parallel to the preparation of the binder system, TCO dispersions were prepared with a nanoparticle concentration of 12.5 wt.% either using an ultrasonic lab homogenizer (in the case of powders) or diluted (in the case of the ready-made dispersion). In the case of nanopowders, the powders were dispersed by adding 4 g of the TCO powder into the mentioned dispersant medium (cf. Table 1). The dispersions were subsequently treated with a sonotrode (UP 200S, Hielscher Ultrasound Technology, Germany, equipped with a sonotrode Micro Tip S3) for 30 min and particle sizes were investigated by dynamic light scattering (DLS) using a Malvern Zetasizer afterwards. The resulting dispersions were processed timely with the above-mentioned sols.

#### 4.2.3. Preparation of the Coating Dispersion

The coating dispersion was prepared by mixing the binder system and the TCO dispersion before stirring it for at least 10 min with a magnetic stirrer. The share of TCO in the resulting coating composite was adjusted by adding specific amounts of the METHA sol. The share of TCO is given in wt.% in the results and calculated by assuming a theoretically complete condensation of the METHA during curing of the composite.

#### 4.2.4. Spin Coating of the Coating Dispersion

Coating was carried out using a custom-made spin-coater that allowed us to prepare samples with a diameter of 80 mm. Circular samples with a diameter of 80 mm were cut (PET films and roofing membranes). The samples were fixed on the spin-coater with double-sided tape. Two milliliters of the sols was applied dropwise. After application, the spin-coater ran for an additional 60 s. The spin-coater was used at 1000 rpm. The coatings were dried and cured in a lab oven at 130 °C for at least 30 min.

### 4.3. Analytics

Scanning electron microscopy (SEM) was conducted using a Hitachi S3400N (Hitachi, Feldkirchen, Germany). The samples were sputtered with gold. UV-VIS-NIR spectra (reflection or transmission) in a range from 200 nm–2.5 µm were recorded with a Perkin-Elmer Lambda 960s equipped with an integrated sphere. Emissivity was measured using a TIR 100-2 of Inglas, Bermatingen, Germany. The spectral range covers 2.5 µm to 40 µm. The accuracy of this device is +/− 0.005. Particle sizes were measured by dynamic light scattering using a Malvern Zetasizer. To visualize heat reflection, a FLIR-T355 IR camera was used (spectral range 7.5–13 µm, sensitivity: 50 mK). The scaling of the temperature range in the thermograms cannot be adjusted on the measuring device. Surface resistance was measured according to DIN EN 1149-1 using a ring electrode and a Teraohmmeter Resistomat Type 2408 of Burster. Sheet resistance was measured with a four-point probe by Jandel, Leighton Buzzard, UK, Model RM3-AR. Before measuring resistances, the samples were conditioned for 24 h at 20 °C and 65% rel. humidity.

The UV-VIS-NIR spectra covering a range from 200 nm to 22.5 µm were measured in an external laboratory (CHT Germany GmbH, Tübingen, Germany).

## Figures and Tables

**Table 1:** Summary of TCO, dispersant medium, mean particle size (DLS), optical appearance and polydispersity index (PDI) of the three most promising dispersions in view of stability and particle size.

	Received as	Dispersant Medium	Supplier	Appearance of the Dispersion	Average Particle Size (nm)	PDI
ITO	Dispersion	Isopropanol	Sigma-Aldrich	Blue	60	0.128
ITO	Powder	Isopropanol	Sigma-Aldrich	Grey	245	0.211
AZO	Powder	2-Methoxyethanol	IoLi-Tec	Grey	1290	0.636

**Table 2:** Sheet resistance and emissivity values for PET films coated with composite coatings prepared with different TCOs (n.m. = not measurable).

Sample	Sheet Resistance	Emissivity
	mOhm/sq	
Uncoated	n.m.	0.8695
ITO dispersion	330	0.7118
ITO powder	330	0.6780
AZO powder	n.m.	0.8290
